# Molecular Profiling of Foodborne Pathogens in Ready-to-Eat Foods, Al-Madinah Al-Munawarah, Saudi Arabia

**DOI:** 10.3390/biology15010104

**Published:** 2026-01-05

**Authors:** Omar Almutairi, Ihab M. Moussa, Eman Marzouk, Adil Abalkhail, Ayman Elbehiry

**Affiliations:** 1 Department of Botany and Microbiology, College of Science, King Saud University, P.O. Box 2455, Riyadh 11451, Saudi Arabia; 442106722@student.ksu.edu.sa (O.A.); imoussa1@ksu.edu.sa (I.M.M.); 2 Department of Public Health, College of Applied Medical Sciences, Qassim University, P.O. Box 6666, Buraydah 51452, Saudi Arabia; e.marzouk@qu.edu.sa (E.M.);

**Keywords:** foodborne pathogens, detection, antibiotic resistance, ready-to-eat foods, MALDI-TOF MS, PCR, One Health

## Abstract

Foodborne pathogens remain a global public health concern, and antimicrobial resistance increases their impact. In mass-gathering cities such as Al-Madinah Al-Munawarah, contaminated ready-to-eat (RTE) fast foods can contribute to both local transmission and international spread. In this study, 300 RTE fast food samples, including shawarma, burgers, fried chicken, sandwiches, and salads, were collected from international franchises, local restaurants, and street vendors. Pathogens were identified using conventional culture combined with matrix-assisted laser desorption ionization time-of-flight mass spectrometry (MALDI-TOF MS). Antimicrobial susceptibility testing followed CLSI guidelines, and real-time PCR confirmed species identity and screened resistance determinants. Principal component analysis (PCA) and dendrogram clustering were used to assess diagnostic discrimination. Among the 300 samples, 129 (43.0%) were culture positive. The most common pathogens were *Staphylococcus aureus* (14.3%) and *Escherichia coli* (13.0%), followed by *Salmonella* spp. (9.0%) and *Acinetobacter baumannii* (6.7%). About 35% of *S. aureus* isolates were methicillin resistant (MRSA), and 85% of *A. baumannii* carried OXA-type carbapenemase genes. MALDI-TOF MS achieved 96.1% score-based identification and, with PCA, showed strong interspecies separation. PCR confirmed species identity and detected widespread resistance genes, with genotype–phenotype concordance of at least 80%. Overall, 60.5% of isolates were multidrug resistant. RTE fast foods in Al-Madinah represent reservoirs of MDR pathogens, including carbapenemase-producing *A. baumannii*. The combined use of MALDI-TOF MS and real-time PCR established a rapid and scalable workflow that provided reliable identification and resistance profiling in less than 24 h, compared with 48 to 72 h for conventional methods. This approach supports One Health surveillance in high-risk food settings and strengthens preparedness for mass gatherings.

## Simple Summary

Ready-to-eat (RTE) foods are eaten every day by many people, but they can sometimes contain harmful bacteria if hygiene is not well controlled. This issue is especially important in Al-Madinah Al-Munawarah, where large numbers of visitors gather throughout the year. In this study, we examined common fast foods to look for bacteria linked to food poisoning and to check whether they were resistant to antibiotics. We found several bacteria of public health concern, including *Staphylococcus aureus*, *Escherichia coli*, and *Salmonella*. Some of these bacteria were resistant to more than one antibiotic. We also detected *Acinetobacter baumannii*, a bacterium usually linked to healthcare settings, suggesting possible gaps in food handling practices. Faster laboratory methods helped us identify bacteria more quickly than traditional testing. Our findings highlight the importance of good hygiene and regular monitoring of RTE foods to protect public health, especially during large gatherings.

## 1. Introduction

Foodborne pathogens remain a major global health challenge, causing an estimated 600 million illnesses and 420,000 deaths each year. Children under five years are disproportionately affected and account for about 30 percent of foodborne deaths [1]. Beyond acute gastroenteritis, foodborne infections can lead to severe complications such as septicemia, meningitis, hemolytic uremic syndrome, and chronic gastrointestinal disorders. Among the most concerning bacterial pathogens are *Escherichia coli* (*E. coli*), *Salmonella* spp., *Staphylococcus aureus* (*S. aureus*), *Listeria monocytogenes* (*L. monocytogenes*), and *Acinetobacter baumannii* (*A. baumannii*) because of their high prevalence, virulence, and ability to acquire multidrug resistance (MDR) [2,3,4]. Although *A. baumannii* is not regarded as a classical foodborne pathogen, it has been increasingly detected in food products and food handling environments, where its pronounced environmental persistence and its role as a reservoir of clinically important antimicrobial resistance genes raise growing concerns for food safety and public health within a One Health framework [5,6,7].

The global rise of antimicrobial resistance (AMR) has intensified the risks associated with foodborne pathogens. Many infections that were once treatable have become difficult to manage because of mechanisms such as extended spectrum beta lactamase (ESBL) production and methicillin resistance. These mechanisms were previously restricted to clinical settings but are now frequently reported in food, animal, and environmental sources [8,9,10]. This expansion highlights the need for surveillance systems that follow the One Health framework, which recognizes the link between human, animal, and environmental health in addressing AMR [11].

Fast food, a rapidly expanding part of the global food industry, presents specific challenges for food safety. Large scale production, short preparation times, and complex handling chains increase its vulnerability to contamination. Studies have shown that ready-to-eat (RTE) foods such as sandwiches, shawarma, and fried products can harbor *E. coli*, *S. aureus*, and *Salmonella* spp. at levels that pose significant risks to consumers [12,13]. In Saudi Arabia, dietary habits have shifted in recent decades, with fast food becoming a dominant component of the diet, especially among young adults in urban areas [14]. A recent cross sectional study of more than 600 adolescents in Jeddah and Madinah reported that 28.5% consumed fast food more than twice each week, with higher consumption among males and individuals from higher income families [15].

Despite this nutritional shift, systematic investigations of AMR foodborne pathogens in fast food outlets remain limited. A recent study in Al Qassim Province examined 300 fast food samples, including shawarma and chicken burgers, using proteomic methods. The study detected high prevalence levels of *E. coli* (34%) and *S. aureus* (31%) and identified resistance genes such as *blaTEM*, *blaZ*, and *mecA* [16]. However, similar data are lacking for Al-Madinah Al-Munawarah, a city of major religious significance that receives millions of international visitors each year. This diverse demographic context means that local food safety issues may have international public health consequences.

Traditional microbiological culture and biochemical assays remain essential for detecting foodborne bacteria. However, these methods are labor intensive, time consuming, and limited in discriminatory power. Matrix-assisted laser desorption ionization time of flight mass spectrometry (MALDI TOF MS) has emerged as a transformative tool in clinical and food microbiology. By analyzing species-specific protein spectra, MALDI TOF MS provides a high species-level identification rate, is cost effective, and achieves precise bacterial identification within minutes [17,18]. Its capability can be expanded through principal component analysis (PCA) and dendrogram clustering, which offer epidemiological insights into relatedness among isolates [19].

When combined with antimicrobial susceptibility testing (AST) and molecular assays, MALDI TOF MS serves as a strong complementary platform for foodborne pathogen surveillance. Its ability to deliver rapid and reliable identification has been demonstrated in food safety investigations and has reliably identified pathogens such as *E. coli*, *S. aureus*, and *Salmonella* spp. from RTE foods [16,20,21]. This proteomic and molecular approach supports One Health-based monitoring of AMR circulation in food chains.

In the Middle East and North Africa (MENA) region, several studies have reported high levels of AMR among foodborne pathogens. MDR *E. coli* has been commonly isolated from street-vended meals in Egypt [22], and resistant *S. aureus* has been identified in RTE snacks and sandwiches in Iran [23]. However, in Saudi Arabia, and especially in Al-Madinah Al-Munawarah, such systematic studies remain scarce, creating a critical knowledge gap. Given the city’s role as a center of mass gatherings and transient populations, contamination of fast foods with AMR pathogens may present both local and global risks through potential dissemination by international visitors.

To address this gap, the present study investigated the prevalence, AMR patterns, and molecular characteristics of major foodborne pathogens isolated from RTE foods in Al-Madinah Al-Munawarah. This study combined conventional culture, proteomic identification by MALDI TOF MS, phenotypic AST, and polymerase chain reaction (PCR)-based detection of resistance and virulence genes. To our knowledge, this is the first report from the region to apply MALDI TOF MS for the characterization of foodborne bacteria from fast foods, together with molecular screening of resistance determinants such as *blaTEM*, *blaSHV*, *blaZ*, and *mecA*.

The findings are expected to offer important insights into the microbiological safety of fast foods, clarify their role in AMR dissemination, and support the need for One Health-based surveillance. The results may guide policymakers, public health authorities, and food safety regulators in Saudi Arabia and in other countries on strengthening monitoring systems and implementing effective interventions against MDR foodborne pathogens.

## 2. Materials and Methods

### 2.1. Ethical Approval

This study focused only on RTE food samples collected from retail outlets, restaurants, and street vendors in Al-Madinah Al-Munawarah. No human participants, animals, or personal data were involved. Institutional ethical approval was therefore not required. Because sampling consisted of purchasing food items from publicly accessible outlets under normal consumer conditions, municipal permission was not required under local regulations. All procedures followed Saudi food safety and sampling guidelines.

### 2.2. Study Area, Sampling Frame, and Eligibility Criteria

This cross-sectional study was conducted in Al-Madinah Al-Munawarah, Saudi Arabia. The city hosts millions of pilgrims each year and is a critical setting for the potential spread of antimicrobial resistant pathogens [24,25]. Sampling was conducted between January and September 2024 to account for seasonal variation and to collect foods under routine service conditions.

A stratified random sampling design ensured representation across three food service types: international fast food franchises, locally operated restaurants, and informal or semi-formal street vendors. Stratification is recommended in food safety surveillance to reduce bias toward one outlet type and to capture exposure risks across different socioeconomic groups [26,27]. Specifically, 75 ready-to-eat food samples were collected from international fast food franchises, 75 from locally operated restaurants, and 150 from informal or semi-formal street vendors. This distribution was used to ensure balanced representation across the three sampling strata. Outlets were selected at random from municipal registries, and street vendors were identified through systematic surveys of high-density commercial areas. Sampling occurred during alternating lunch (12:00 to 15:00) and dinner (18:00 to 22:00) periods to capture peak food preparation activity when contamination risks are highest [28].

A total of 300 RTE fast food items were collected, including chicken and beef shawarma, chicken and beef burgers, fried chicken, sandwiches, and salads. Each item (25 to 50 g) was purchased anonymously under standard customer conditions using sterile utensils and gloves to prevent post-purchase contamination. Samples were sealed in sterile stomacher bags, labeled with outlet identification, date, time, and food type, and transported in insulated carriers maintained at 4 ± 2 °C. Laboratory processing began within 2 to 4 h of purchase according to international standards for microbiological food sampling [29].

Inclusion criteria were as follows: RTE foods at the point of sale; items prepared on site during normal operations; samples purchased within Al Madinah municipal limits; portions of at least 25 g; and intact containers during transport. Exclusion criteria were as follows: raw or partially cooked foods; industrially packaged shelf stable products; visibly spoiled or temperature abused items (≥10 °C during transport); duplicate samples from the same batch (limited to two items per outlet per visit and visits spaced by at least seven days); and samples exceeding the 4 h transport limit or failing labeling requirements. All sampling procedures complied with local regulations. The use of explicit criteria supports reproducibility and aligns with best practices in foodborne pathogen surveillance [30,31].

### 2.3. Microbiological Analysis

Approximately 25 g of each sample was aseptically weighed and placed into a sterile stomacher bag with 225 mL of buffered peptone water (BPW; Oxoid Ltd., Altrincham, UK), following the standard 1:10 ratio. Homogenization was performed using a Stomacher blender (Seward Ltd., Worthing, UK) for 60 s. Homogenates were incubated at 37 °C for 18 to 24 h to allow recovery of sublethally injured bacteria.

After pre enrichment, 0.1 mL aliquots were streaked onto selective and differential media: MacConkey agar (Oxoid Ltd., Altrincham, UK) for *E. coli* and other Gram-negative bacilli including *A. baumannii*; Baird Parker agar (Oxoid Ltd., Altrincham, UK) for *S. aureus*; and Xylose Lysine Deoxycholate (XLD) agar (Oxoid Ltd., Altrincham, UK) for *Salmonella* spp. Plates were incubated aerobically at 37 °C for 18 to 24 h. Colonies with typical morphologies were subcultured on nutrient agar to obtain pure isolates.

Representative isolates were examined by Gram staining and routine biochemical assays, including catalase, oxidase, and coagulase tests. Oxidase-negative, non-lactose-fermenting colonies recovered from MacConkey agar were retained for further analysis as Gram-negative isolates of interest. These characteristics were used solely for preliminary screening and were not intended for species-level identification or interpretation of epidemiological relevance. Definitive identification was achieved using MALDI-TOF MS (Bruker Daltonics GmbH, Bremen, Germany, FlexControl software version 3.1) and confirmed by 7500 Fast Real-Time PCR System (Applied Biosystems, Foster City, CA, USA). This culture-based screening approach is commonly applied in studies of multidrug-resistant bacteria detected in food and food-related environments [2,32,33,34].

The microbiological workflow followed a clear sequence of screening, identification, and confirmation. An initial non-selective enrichment step was used to help recover bacteria that may be present in low numbers or under stress in ready to eat foods. Selective and differential media were then used to screen for target bacteria based on their typical colony appearance. Presumptive isolates were identified using MALDI TOF MS, and species identity was confirmed by real-time PCR. This workflow was chosen to reflect routine food safety testing practices and to allow comparison with commonly used laboratory methods. We acknowledge that using a single enrichment step with selective plating may reduce the detection of some pathogens, particularly *Salmonella*, when compared with more complex enrichment protocols. As a result, the findings represent contamination detectable under routine testing conditions rather than complete recovery of all possible pathogens.

For the prevalence analysis, each food sample was treated as a single unit. A sample was recorded as positive when at least one isolate of a given bacterial species was detected. More than one bacterial species could be recovered from the same food sample. For further testing, including antibiotic susceptibility, molecular analysis, and MALDI-TOF MS profiling, one representative isolate per bacterial species from each sample was selected to prevent repeated counting of the same source. Prevalence values were calculated using the total number of food samples examined.

### 2.4. Protein Extraction and MALDI-TOF MS Identification

Pure colonies were processed using the ethanol and formic acid extraction method recommended by Bruker Daltonics (Bremen, Germany) [35]. One to two fresh colonies were suspended in 300 µL sterile water and mixed with 900 µL absolute ethanol. After centrifugation at 13,000× *g* for 2 min, the supernatant was discarded and then the pellet was air dried. The pellet was resuspended in 30 µL of 70% formic acid and then mixed with 30 µL of acetonitrile. After centrifugation, 1 µL of the clear supernatant was spotted in duplicate onto a polished steel target plate. Each spot was overlaid with 1 µL of α cyano 4 hydroxycinnamic acid (HCCA) matrix and allowed to dry.

Spectra were acquired using a Bruker Microflex LT mass spectrometer (Bruker Daltonics GmbH, Bremen, Germany) operating in linear positive ion mode with an *m*/*z* range of 2000 to 20,000 Da. Calibration and quality control used the Bruker Bacterial Test Standard (BTS, *E. coli* DH5α). Spectra were analyzed using Bruker Biotyper software (version 3.0) and the current Bruker reference library. Identification scores ≥ 2.0 indicated species-level identification, while scores of 1.7 to 1.99 were accepted at the genus level [36,37]. Non-pathogenic or environmental bacteria identified by MALDI-TOF MS were recorded but excluded from downstream analyses because this study focused exclusively on major foodborne pathogens.

PCA and dendrogram analyses were used only to visualize overall similarity among MALDI-TOF MS spectra. For each isolate, spectra were generated from repeated technical measurements and processed using standard software settings, including baseline correction, smoothing, and normalization. Clustering was based on spectral similarity and was applied to compare patterns among isolates. These analyses are descriptive and reflect similarity at the species level. They do not indicate strain relatedness, transmission, or epidemiological linkage, as higher-resolution typing methods such as whole-genome sequencing or MLST were not performed.

### 2.5. Antimicrobial Susceptibility Testing

AST was performed using the Kirby Bauer disk diffusion method on Mueller Hinton agar (Oxoid Ltd., Altrincham, UK) according to CLSI performance standards [38]. The antibiotic panel included agents relevant to foodborne pathogens in clinical and veterinary contexts.

Beta lactam antibiotics included ampicillin (10 µg), cefoxitin (30 µg), and cefotaxime (30 µg). Cefoxitin was used as a surrogate marker for methicillin resistance in *S. aureus* [38,39,40]. Aminoglycosides included gentamicin (10 µg) and streptomycin (10 µg). Tetracyclines included tetracycline (30 µg) and doxycycline (30 µg). Fluoroquinolones included ciprofloxacin (5 µg) and levofloxacin (5 µg). Additional antibiotics included chloramphenicol (30 µg), trimethoprim sulfamethoxazole (1.25/23.75 µg), and erythromycin (15 µg) for staphylococci.

Antibiotic panels were adjusted for each bacterial group. For *S. aureus*, the panel included ampicillin, cefoxitin, tetracycline, erythromycin, ciprofloxacin, chloramphenicol, and trimethoprim sulfamethoxazole. For Enterobacterales (*E. coli* and *Salmonella* spp.), the panel included ampicillin, cefotaxime, tetracycline, streptomycin, ciprofloxacin, levofloxacin, chloramphenicol, and trimethoprim sulfamethoxazole. For *A. baumannii*, the panel included gentamicin, tetracycline, doxycycline, and ciprofloxacin. Cefoxitin was used only for *S. aureus*.

Bacteria were adjusted to a 0.5 McFarland standard and inoculated onto Mueller Hinton agar plates. Disks were applied aseptically, and plates were incubated at 35 ± 2 °C for 16 to 18 h. Inhibition zones were measured and interpreted using CLSI breakpoints.

Quality control included *S. aureus* ATCC 25923 and *E. coli* ATCC 25922. All tests were performed in duplicate. Discordant results were repeated. MDR was defined as non-susceptibility to at least one agent in three or more antimicrobial classes [39].

### 2.6. Molecular Detection of Antimicrobial Resistance Genes

Real-time polymerase chain reaction (RT PCR) was used for species confirmation and detection of AMR genes. Assays were performed using an Applied Biosystems 7500 Fast RT PCR System (Applied Biosystems, Foster City, CA, USA). Genomic DNA was extracted using the QIAamp DNA Mini Kit (Qiagen, Hilden, Germany). DNA purity and concentration were measured using a NanoDrop spectrophotometer (Thermo Fisher Scientific, Wilmington, DE, USA).

Species-specific markers included *uidA* (β-glucuronidase) for *E. coli*, *nuc* (thermonuclease) for *S. aureus*, *invA* (invasion gene) for *Salmonella* spp., and *blaOXA-51*-like for *A. baumannii*. The *invA* assay confirms *Salmonella enterica* at the species level but does not differentiate serovars; classical serotyping was not performed in this study, and therefore serovar assignment was not determined. Amplification conditions were optimized for each primer set, with melt-curve analysis used to verify specificity. This molecular step strengthened the reliability of MALDI-TOF MS results and minimized misidentification.

Resistance genes screened included *blaTEM*, *blaCTX M*, and *blaSHV* in Enterobacterales; *blaZ* and *mecA* in staphylococci; and *blaOXA 23*, *blaOXA 24*/*40*, and *blaOXA 58* in *A. baumannii*. Additional targets included *tetA* and *tetM* (tetracyclines); *aac(6′) Ib* and *aph(3′) IIIa* (aminoglycosides); *ermB* (macrolides); and *sul1* (sulfonamides).

**Figure 1:**
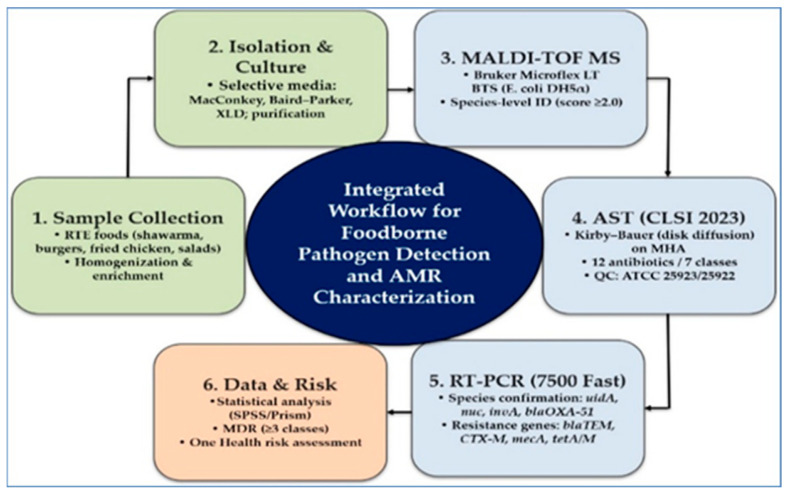
Integrated workflow for foodborne pathogen detection and AMR characterization. The stepwise process includes sample collection, bacterial isolation and culture, species identification by MALDI-TOF MS, antimicrobial susceptibility testing [38], and molecular confirmation of species and resistance genes, followed by data analysis and risk assessment.

PCR reactions (20 µL) contained 10 µL SYBR Green Master Mix, 0.5 µM primers, and a 2 µL DNA template. Cycling conditions were 95 °C for 10 min, followed by 40 cycles of 95 °C for 15 s and 60 °C for 1 min. Negative and positive controls were included. A cycle threshold (Ct) ≤ 35 with a corresponding melt peak was considered positive. Primer sequences are listed in Table 1. This combined workflow provided dual confirmation of species identity and resistance profiles. MALDI TOF MS enabled rapid identification, while RT PCR verified species and detected AMR genes. Figure 1 presents an overview of the workflow.

### 2.7. Data Analysis

Data were analyzed using SPSS Statistics version 26.0 (IBM Corp., Armonk, NY, USA). Descriptive statistics summarized pathogen prevalence, AMR patterns, and resistance gene frequencies. MDR was defined as non-susceptibility to at least one agent in three or more antimicrobial classes [39]. Comparisons of resistance rates across bacterial species and food categories used the Chi square test or Fisher exact test. A *p* value <0.05 was considered statistically significant.

## 3. Results

### 3.1. Sample Characteristics and Food Categories

A total of 300 RTE fast food items were collected from outlets across Al-Madinah Al-Munawarah. Sampling was stratified to represent different food service types. International fast food franchises contributed 75 samples (25%), local restaurants contributed 75 samples (25%), and informal or semi-formal street vendors contributed 150 samples (50%).

The food categories reflected the most frequently consumed fast foods in the region. These categories included chicken shawarma (40 samples, 13.3%), beef shawarma (40 samples, 13.3%), burgers including chicken and beef (50 samples, 16.7%), fried chicken (50 samples, 16.7%), sandwiches other than shawarma or burgers (60 samples, 20.0%), and fresh salads (60 samples, 20.0%). The distribution of samples by outlet type and food category is shown in Table 2.

### 3.2. Bacterial Isolation and Primary Identification

Of the 300 RTE fast food samples, 129 samples (43.0%) were culture-positive on selective and differential media. Preliminary identification based on colony morphology, Gram staining, and biochemical assays indicated the presence of *E. coli*, *S. aureus*, *Salmonella* spp., and *A. baumannii*.

*S. aureus* was the most common isolate (43 of 300; 14.3%), followed by *E. coli* (39 of 300; 13.0%), *Salmonella* spp. (27 of 300; 9.0%), and *A. baumannii* (20 of 300; 6.7%). Contamination patterns differed by food type. Shawarma and burgers were more often contaminated with *E. coli* and *S. aureus*, while fried chicken and salads were more frequently associated with *Salmonella* spp. and *A. baumannii*. The distribution of isolates across food categories is presented in Table 3.

### 3.3. MALDI-TOF MS Identification

All presumptive isolates obtained through culture and biochemical tests were analyzed by MALDI TOF MS. Protein spectra generated by the Bruker Microflex LT system showed clear and reproducible peaks within the 2000 to 20,000 Da range. Identification scores were interpreted using Bruker Biotyper criteria, with values ≥ 2.0 accepted for species level identification and scores of 1.7 to 1.99 accepted at the genus level. MALDI-TOF MS successfully identified the majority of isolates at the species level, with the remaining isolates achieving reliable genus-level identification, in accordance with Bruker Biotyper score criteria. Overall identification showed high concordance with conventional methods. Because MALDI TOF MS and *invA* PCR confirm *Salmonella enterica* only at the species level, serovar determination was not performed. All isolates are therefore reported as *Salmonella* spp. in this study.

In addition to these four targeted foodborne pathogens, MALDI-TOF MS also detected several non-pathogenic or low-risk bacterial species, including *Micrococcus luteus*, *S. xylosus*, *Kocuria kristinae*, *Lactobacillus plantarum*, *Leuconostoc mesenteroides*, *Bacillus subtilis*, and *Hafnia alvei*. These organisms are commonly associated with food matrices and environmental microbiota and do not represent recognized foodborne hazards. Because the aim of this work was to characterize clinically important pathogens and their AMR patterns, these non-pathogenic isolates were documented but excluded from all downstream analyses, including prevalence reporting, AST, PCR genotyping, PCA, and dendrogram clustering.

In total, 124 of 129 isolates (96.1%) achieved species-level identification, and 5 isolates (3.9%) were identified at the genus level. Most *S. aureus* and *E. coli* isolates produced secure species-level scores. A small number of *Salmonella* spp. and *A. baumannii* isolates yielded genus-level scores, likely reflecting intraspecies spectral variation. Identification scores by species are summarized in Table 4.

**Figure 2:**
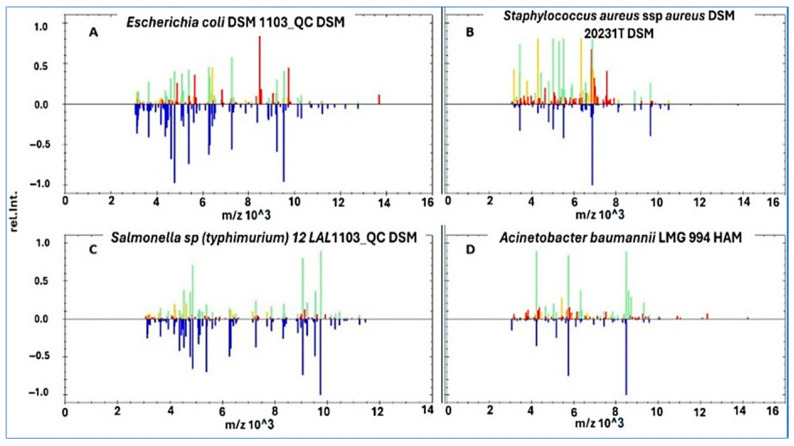
Representative MALDI-TOF MS peak intensity spectra of four bacterial isolates recovered from RTE fast foods. (**A**) *E. coli*; (**B**) *S. aureus*; (**C**) *Salmonella* spp.; and (**D**) *A. baumannii*. Blue peaks in the lower part of each spectrum represent the deposited reference spectra used for pattern matching, while green peaks in the upper part indicate well-matched signals; red and yellow peaks denote mismatched and intermediate matches, respectively. Each spectrum shows characteristic protein mass peaks within the 2000–20,000 Da range, reflecting species-specific fingerprints that enabled reliable identification. The reproducibility of these spectra underscores the robustness of MALDI-TOF MS for rapid foodborne pathogen detection.

Representative MALDI TOF MS spectra are shown in Figure 2. Panel A presents *E. coli*, which displayed dominant peaks between 5000 and 7000 Da, consistent with ribosomal protein signatures. Panel B shows *S. aureus*, with high intensity peaks detected between 3500 and 7000 Da. Panel C illustrates *Salmonella* spp., characterized by a broader distribution of peaks across 3000 to 9750 Da. Panel D depicts *A. baumannii*, which showed distinct spectral clusters between 4500 and 8500 Da. These species-specific fingerprints demonstrate the discriminatory capacity of MALDI TOF MS and support its agreement with culture-based identification. The spectral variability observed across isolates also provides qualitative support for subsequent clustering analyses.

**Figure 3:**
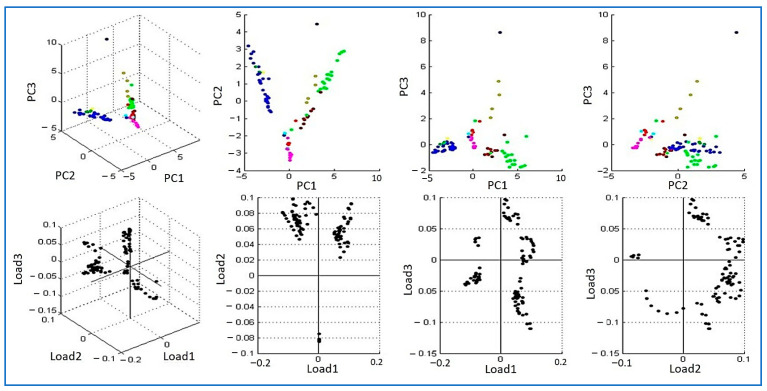
Principal component analysis (PCA) of MALDI-TOF MS spectra from 129 bacterial isolates recovered from RTE fast foods. The three-dimensional scatter plot with corresponding two-dimensional projections (PC1–PC3) shows distinct species-specific clustering. Different colors represent different bacterial species included in the analysis. The figure was generated directly using the Microflex LT system, and the axis labeling and numeric scaling reflect the default output format of the instrument.

To further assess discriminatory performance, principal component analysis (PCA) was applied to spectra from all 129 isolates, as shown in Figure 3. Both three dimensional and two-dimensional scatterplots demonstrated clear species-specific clustering. *E. coli* and *S. aureus* formed compact and well-separated groups. *Salmonella* spp. displayed a wider distribution, consistent with the diversity of this genus. *A. baumannii* formed a distinct cluster driven by high-mass spectral features. Loading plots indicated the peaks contributing most to interspecies variance. These results confirm that MALDI TOF MS combined with PCA provides reliable species level identification and supports epidemiological profiling.

**Figure 4:**
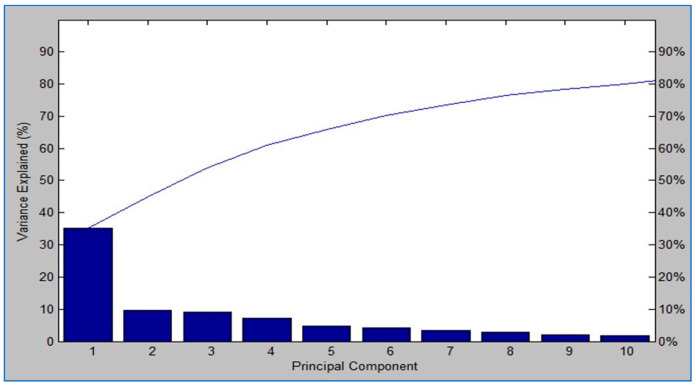
Scree plot of PCA based on MALDI-TOF MS spectra from 129 bacterial isolates recovered from RTE fast foods. PC1 (~35%), PC2 (~15%), and PC3 (~10%) together explained >60% of the variance, while later components contributed minimally. This variance structure confirms that species-level discrimination can be achieved using only the first three components, supporting the integration of MALDI-TOF MS into rapid, high-throughput diagnostic workflows.

The scree plot shown in Figure 4 summarizes the variance explained by principal components. The first component accounted for about 35% of total variance, followed by the second (about 15%) and the third (about 10%). The first three components together captured more than 60% of the variance. Higher components contributed only marginally. By the tenth component, nearly 80% of the total variance was explained. These findings validate the stability of the PCA model and confirm that the first three components were sufficient for species level discrimination.

**Figure 5:**
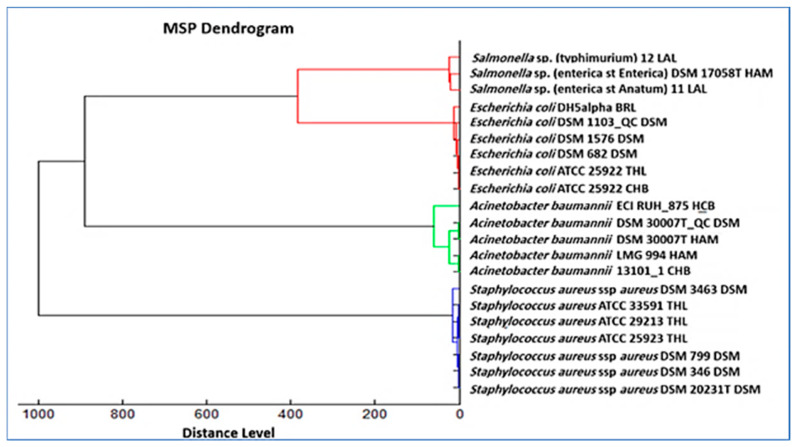
MSP dendrogram of MALDI-TOF MS data showing clustering of field isolates with reference strains. Four distinct clusters were resolved: *Salmonella enterica* (clustering with reference strains labeled Typhimurium, Enteritidis, and Anatum; serovar labels are inferred from reference clustering and were not confirmed by classical serotyping), *E. coli* (including ATCC 25922, DSM 1103, DSM 1576, and DSM 682), *A. baumannii* (DSM 30007T, LMG 994, and DSM 13101), and *S. aureus* (ATCC 25923, ATCC 29213, ATCC 33591, DSM 3463, DSM 20231, and DSM 799). Short branch lengths confirmed intraspecies reproducibility, while long branch distances reflected interspecies divergence.

A main spectrum profile (MSP) dendrogram was constructed using the field isolates together with reference strains (Figure 5). Four distinct clusters were observed, corresponding to the species identified in this study. Because classical serotyping was not performed, any serovar names shown in the dendrogram represent inferred similarity to reference strains and should not be interpreted as confirmed serovars. *E. coli* isolates aligned closely with quality control strains. *A. baumannii* isolates clustered with established reference strains, confirming their spectral reproducibility. *S. aureus* isolates grouped with ATCC and DSM reference strains, supporting identification reliability. Short branch lengths within clusters reflected intraspecies similarity, and long branch distances between groups indicated proteomic divergence among species.

### 3.4. Antimicrobial Susceptibility Testing (AST) Results

All confirmed isolates (n = 129) were tested for antimicrobial susceptibility using the Kirby Bauer disk diffusion method. Resistance patterns varied by species, as shown in Table 5, and were common across several antibiotic classes.

*S. aureus* isolates (n = 43) showed high resistance to ampicillin (72.1%) and erythromycin (62.8%). Moderate resistance was observed to tetracycline (44.2%) and ciprofloxacin (39.5%). Resistance to cefoxitin, used as a surrogate marker for methicillin resistance, was detected in 34.9% of isolates, confirming the presence of MRSA. All *S. aureus* isolates were susceptible to chloramphenicol.

*E. coli* isolates (n = 39) demonstrated high resistance to ampicillin (69.2%) and trimethoprim sulfamethoxazole (61.5%). Resistance to cefotaxime was detected in 43.6% of isolates, suggesting the presence of ESBL producers. Fluoroquinolone resistance ranged from 33.3% for ciprofloxacin to 30.8% for levofloxacin.

*Salmonella* spp. (n = 27) showed lower resistance overall but remained a concern. Resistance was observed to ampicillin (40.7%), tetracycline (29.6%), and streptomycin (25.9%). All isolates were susceptible to chloramphenicol and levofloxacin.

*A. baumannii* (n = 20) exhibited the highest levels of phenotypic resistance among the bacterial species examined. High resistance rates were observed to tetracycline and ciprofloxacin (each >70%), while gentamicin resistance was detected in 55% of isolates and doxycycline resistance in 45%. Ampicillin and cefotaxime were not included in the resistance analysis for *A. baumannii* due to intrinsic resistance and were therefore not considered in the assessment of acquired MDR.

Overall, 78 of the 129 isolates (60.5%) met the definition of MDR, being resistant to at least one agent in three or more antimicrobial classes. MDR prevalence was highest in *A. baumannii* (85.0%), followed by *S. aureus* (65.1%), *E. coli* (61.5%), and *Salmonella* spp. (37.0%).

Statistical analysis showed significant differences in resistance prevalence across species. Resistance to beta lactams, including ampicillin and cefotaxime, was significantly higher in *A. baumannii* and *E. coli* compared with *Salmonella* spp. (*p* < 0.01). *S. aureus* showed significantly greater resistance to erythromycin (*p* < 0.05). MDR prevalence was significantly higher in *A. baumannii* than in all other species (*p* < 0.001).

These findings have diagnostic relevance. Cefoxitin resistance in *S. aureus* confirms MRSA detection. Cefotaxime resistance in *E. coli* suggests ESBL production. The lower resistance profile of *Salmonella* spp. helps differentiate it from other pathogens. The high MDR burden in *A. baumannii* indicates a potential for carbapenem resistance due to OXA type carbapenemase genes, although phenotypic confirmation requires carbapenem testing. These results support the diagnostic prioritization of foodborne pathogens in high-risk settings.

Ampicillin and cefotaxime were not included in the antimicrobial susceptibility testing panel for Acinetobacter baumannii due to intrinsic resistance and are therefore reported as not applicable (NA).

### 3.5. Distribution of Antimicrobial Resistance Genes

RT PCR assays confirmed the molecular identity of all isolates and supported the MALDI TOF MS results. Species specific markers, including *uidA* for *E. coli*, *nuc* for *S. aureus*, *invA* for *Salmonella* spp., and *blaOXA 51* like for *A. baumannii*, were consistently amplified and provided reliable confirmation of culture based and proteomic identification.

In *E. coli*, *blaTEM* was detected in 56.4% (22/39) of isolates, while *blaCTX-M* and *blaSHV* were detected in 41.0% (16/39) and 30.8% (12/39), respectively, using all *E. coli* isolates as the denominator (Table 6). A significant difference was observed between *blaTEM* and *blaSHV* prevalence (*p* < 0.05). In *S. aureus*, *blaZ* was present in 69.8% (30/43) of isolates, and *mecA* was detected in 32.6% (14/43), consistent with the phenotypic identification of MRSA.

Among *Salmonella* spp., tetracycline and sulfonamide resistance genes were detected, including *tetA* in 33.3% (9/27) and sul1 in 25.9% (7/27) of isolates, and these findings aligned with phenotypic resistance patterns. In *A. baumannii*, *OXA*-type genes were detected at varying frequencies among all isolates (n = 20). *blaOXA-23* was detected in 70.0% (14/20), *blaOXA-24/40* in 45.0% (9/20), and *blaOXA-58* in 25.0% (5/20). The intrinsic *blaOXA-51*-like gene was detected in all isolates (100%, 20/20) and was used as a species confirmation marker rather than as an indicator of phenotypic carbapenem resistance.

Statistical analysis showed strong associations between several resistance genes and phenotypic MDR patterns. In *E. coli*, *blaCTX-M* carriage was significantly associated with reduced susceptibility to cefotaxime (*p* < 0.01). In *A. baumannii*, *blaOXA-23* carriage was significantly associated with resistance to cefotaxime and ciprofloxacin (*p* < 0.001), consistent with MDR phenotypes; however, these associations do not confirm carbapenemase activity in the absence of carbapenem susceptibility testing. Overall, 71.3% of isolates carried at least one major resistance gene, confirming the widespread genetic basis of MDR across species.

**Figure 6:**
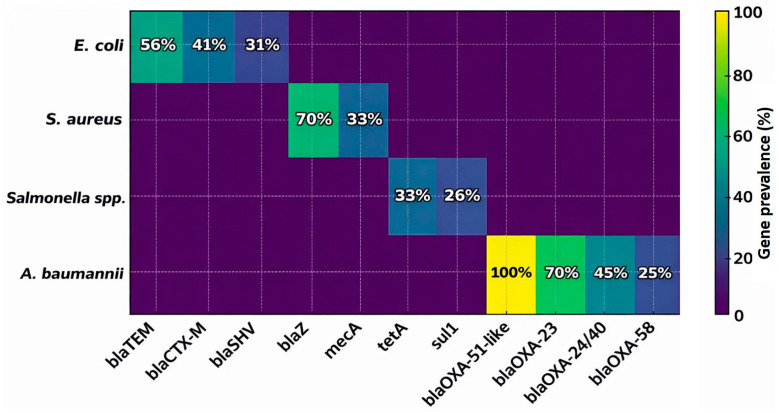
Heatmap of AMR gene prevalence detected by real-time PCR among bacterial isolates from RTE fast foods. Values indicate the percentage of isolates per species carrying each gene. *E. coli* harbored *blaTEM*, *blaCTX-M*, and *blaSHV*; *S. aureus* carried *blaZ* and *mecA*; *Salmonella* spp. showed *tetA* and *sul1*; while *A. baumannii* demonstrated high prevalence of OXA-type carbapenemases (*blaOXA-51*-like, *blaOXA-23*, *blaOXA-24/40*, and *blaOXA-58*).

Table 6 summarizes the distribution of resistance genes and their statistical associations. Figure 6 illustrates the predominance of beta lactamase genes in *E. coli*, methicillin resistance determinants in *S. aureus*, tetracycline and sulfonamide resistance in *Salmonella* spp., and OXA type carbapenemases in *A. baumannii*.

These molecular findings should be interpreted with consideration of the testing scope. The detection of *blaCTX-M*, *blaTEM*, and *blaSHV* reflects the presence of ESBL-associated resistance genes but does not constitute phenotypic confirmation of ESBL production. Similarly, detection of *OXA*-type genes in *A. baumannii* does not confirm carbapenemase activity in the absence of carbapenem susceptibility testing.

### 3.6. Correlation Between Phenotypic Resistance and Resistance Genes

Phenotypic resistance profiles obtained from AST were compared with the presence of corresponding resistance genes detected by real-time PCR to assess genotype and phenotype concordance. Concordance (%) was defined as the proportion of isolates in which phenotypic resistance matched the presence or absence of the relevant gene, using all isolates of each species as the denominator.

As shown in Table 7, *E. coli* isolates demonstrated strong associations between beta lactam resistance and the presence of *blaTEM*, *blaCTX-M*, and *blaSHV*, with an overall concordance of 82.0% (*p* < 0.01). *S. aureus* isolates showed excellent agreement between resistance to penicillin and cefoxitin and the presence of *blaZ* and *mecA*, with concordance of 88.0% (*p* < 0.001). In *Salmonella* spp., tetracycline and sulfonamide resistance correlated significantly with *tetA* and *sul1*, resulting in concordance of 84.0% (*p* < 0.05). In *A. baumannii*, the presence of OXA-type genes (*blaOXA-23*, *blaOXA-24/40*, and *blaOXA-58*) was associated with resistance to beta lactams and fluoroquinolones, with concordance of 85.0% (*p* < 0.001); however, this analysis does not assess carbapenem resistance due to the absence of carbapenem testing in the AST panel.

**Figure 7:**
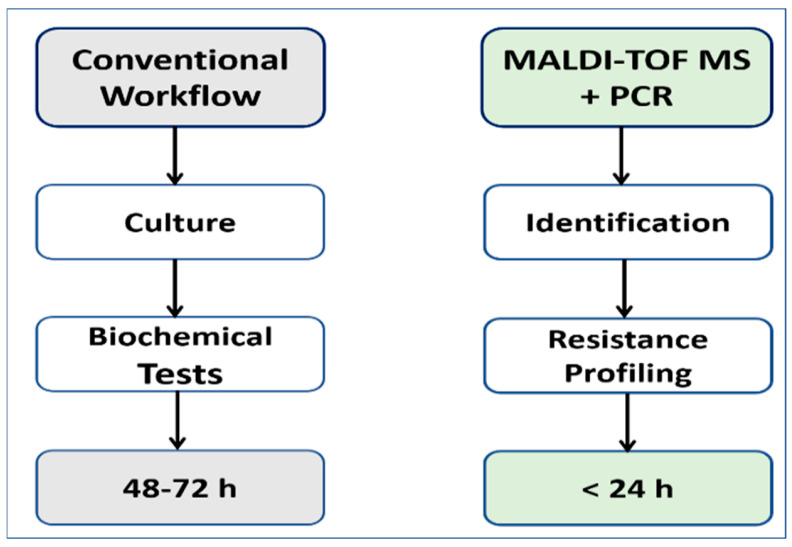
Comparative diagnostic workflows. The conventional approach (culture followed by biochemical testing) requires 48–72 h, whereas the MALDI-TOF MS combined with PCR workflow provides rapid identification and resistance profiling in <24 h. This comparison highlights the diagnostic advantage of MALDI-TOF MS as a fast and reliable tool for foodborne pathogen detection and AMR surveillance.

Across all pathogens, gene carriage showed at least 80% agreement with AST findings, confirming that molecular detection reliably validated phenotypic AMR patterns. As shown in Figure 7, this integrated workflow highlights the practical utility of MALDI TOF MS-based diagnostics for rapid pathogen detection and AMR surveillance.

## 4. Discussion

Foodborne pathogens remain a major global public health threat because they can cause widespread outbreaks and contribute to the growing burden of AMR. Conventional diagnostic methods, including culture and biochemical testing, are reliable but require considerable time, often 48 to 72 h, which can delay clinical and public health responses. In contrast, our findings show that MALDI TOF MS, particularly when combined with PCR-based detection of resistance determinants, offers a rapid and reproducible diagnostic alternative. This integrated workflow provides species-level identification within minutes and delivers clinically useful resistance information in less than 24 h. These advantages highlight its clear benefit over traditional methods. The strong agreement between phenotypic and genotypic findings and the accelerated diagnostic timeline shown in Figure 7 demonstrate the value of this approach for laboratory diagnostics and for One Health surveillance. Because MALDI TOF MS platforms are already available in many clinical and public health laboratories, combining them with PCR represents a scalable and cost-effective strategy that improves diagnostic turnaround and supports outbreak investigations, food safety monitoring, and infection control during mass gatherings.

From a diagnostic perspective, integrating MALDI TOF MS with real-time PCR improved pathogen identification and AMR profiling. MALDI-TOF MS provided rapid identification, achieving species-level classification for most isolates based on established score thresholds, while a small proportion were identified at the genus level. PCR supported taxonomic assignment and resistance gene detection; however, species-level identification rates rather than validated reproducible identification standards are reported, as no independent reference standard was applied. These methods achieved genotype and phenotype concordance of at least 80%. This combined workflow reduced diagnostic turnaround to less than 24 h, compared with 48 to 72 h for conventional culture-based methods. The unified approach strengthens One Health surveillance by enabling timely detection of MDR foodborne pathogens, particularly in high-risk environments such as mass gathering cities.

Although MALDI-TOF MS also identified several non-pathogenic or low-risk bacterial species such as *Micrococcus luteus*, *Staphylococcus xylosus*, *Kocuria kristinae*, *Lactobacillus plantarum*, *Leuconostoc mesenteroides*, *Bacillus subtilis*, and *Hafnia alvei*, these organisms were intentionally excluded from the analysis. They represent naturally occurring food-associated or environmental microbiota and are not considered established foodborne pathogens. In contrast, *A. baumannii*, although not a classical foodborne pathogen, was retained in the analysis because of its recognized role as an opportunistic pathogen and its public health relevance when detected in food and food-handling environments, particularly in the context of AMR. For this reason, only the four clinically important pathogens (*S. aureus*, *E. coli*, *Salmonella* spp., and *A. baumannii*) were included in the prevalence analysis, AST, molecular characterization, PCA, and dendrogram clustering.

The present study demonstrated a substantial burden of foodborne pathogens, with *S. aureus* and *E. coli* predominating, particularly in shawarma and burger samples. This pattern aligns with earlier reports from the region and from other low- and middle-income contexts. A nationwide survey from Algeria detected *S. aureus* in 23.2% of 207 RTE samples and noted contamination in meat and fish based foods, reflecting risks associated with poor handling and post cooking cross contamination [56]. Meta analyses from developing countries report comparable findings, with pooled prevalence estimates of *E. coli* at 23.8% and *Salmonella* spp. at 17.4% in RTE foods [57].

Similar observations have been reported in Egypt, where *S. aureus* was detected in 50.8% of RTE meat sandwiches in Benha, suggesting that vendor type and food category influence contamination risk [58]. In Mansoura, MDR *S. aureus* was isolated from RTE pizzas, indicating that high turnover fast foods may serve as major transmission vehicles [59]. In Saudi Arabia, surveys of shawarma and other fast food outlets have frequently detected *S. aureus*, *E. coli*, and *Salmonella* spp., supporting the national relevance of our findings [60].

Only a few nationally representative studies in Saudi Arabia have used modern diagnostic platforms to characterize AMR patterns in RTE fast foods. A recent study from Al Qassim analyzed 80 RTE items and detected *S. aureus* (27.5%), *E. coli* (32.5%), *A. baumannii* (18.75%), and *Salmonella enterica* (15%) using MALDI TOF MS, AST, and PCR [61]. However, differences in study region, outlet types, and seasonal timing limit direct comparison with the Al Madinah findings. Additional work from Al Ahsa reported *S. aureus* in 11.1% of 90 fast food sandwiches (with about 30% of those identified as MRSA) and *E. coli* in 5.6% of samples, though without extensive AMR genotyping or modern identification methods [62]. Another assessment of 108 cafeteria meals in Makkah documented *S. aureus* counts reaching 9.8 × 10^3^ CFU/mL and *E. coli* contamination in 42% of samples, indicating widespread contamination in cooked or prepared meals. These limited national data highlight a lack of high-resolution, AMR-focused surveillance in RTE foods across Saudi Arabia.

The detection of *blaOXA-51*-like should be interpreted with caution, as this gene is a chromosomally encoded marker that is widely used for species identification within the *A. baumannii* complex rather than as direct evidence of phenotypic carbapenem resistance. Although *blaOXA-51*-like was originally considered intrinsic to *A. baumannii*, later studies demonstrated that related genes can also be detected in non-*baumannii Acinetobacter* species, highlighting its value as a taxonomic marker rather than a standalone indicator of carbapenem resistance. In the absence of carbapenem susceptibility testing, the presence of *blaOXA-51*-like alone does not support conclusions regarding carbapenemase production or clinically relevant carbapenem resistance [44,63].

A notable finding of this study was the detection of *A. baumannii* in 6.7% of samples. This pathogen has traditionally been associated with hospitals but is increasingly reported in food sources. In [64], the authors detected *A. baumannii* in raw chicken meat in China, while [65] recovered it from multiple animal meats in Saudi Arabia, indicating its broad presence in local food supplies. Ababneh et al. [5] also detected *A. baumannii* in vegetables and fruits, many of which showed extensive drug resistance and strong biofilm formation. In the present study, OXA-type genes were detected in *A. baumannii* isolates; however, no phenotypic carbapenem susceptibility testing or molecular typing was performed. Therefore, these findings are reported descriptively and do not allow inference regarding carbapenem resistance, transmission dynamics, or links between community and hospital reservoirs.

The proportion of MRSA in this study (about 35%, based on cefoxitin resistance) also warrants attention. This prevalence is much higher than global estimates, where MRSA in meat products is typically reported at 3% to 4% (3.2% in a 2017 synthesis and 3.72% in a 2025 update) [66,67]. Higher rates have been reported in parts of the Eastern Mediterranean, which aligns with our results and suggests contributions from food handler carriage, poor slicer sanitation, and inadequate hygiene practices [56].

These findings indicate that assembled fast food items such as shawarma and burgers are consistent hotspots for contamination by *S. aureus* and *E. coli*. They also show that staphylococcal burdens in parts of the MENA region exceed global averages and that the detection of *A. baumannii* in RTE foods represents a public health concern when viewed in the context of environmental contamination and AMR. In a mass gathering city such as Al Madinah, even small contamination events could have wide consequences because of international dispersion. The MALDI TOF MS and PCR results therefore support targeted hygiene interventions, including improved slicer sanitation, enhanced handler training, routine screening, and integration of One Health-based surveillance in high-throughput food outlets [57].

Rapid and reliable pathogen identification is essential for interpreting epidemiological patterns and guiding timely interventions. Although culture and biochemical methods remain valuable, they are slow and resource intensive [68]. MALDI TOF MS provides an effective alternative because it offers rapid, cost efficient, and reproducible species-level identification [69,70,71]. In this study, MALDI TOF MS confirmed species identity within minutes, greatly reducing diagnostic turnaround compared with conventional workflows. In addition, PCA and dendrogram-based analyses enabled visualization of relationships among isolates obtained from different outlets. Comparable findings have been reported regionally. Elbehiry et al. [72] achieved 100% reliable identification of *E. coli*, *S. aureus*, *Salmonella enterica*, and *A. baumannii* in Qassim Province, with dendrograms clustering isolates alongside reference strains.

Other studies have extended MALDI TOF MS applications in food safety. Kumar et al. [73] identified *A. baumannii* and other Gram-negative bacteria in meat and environmental samples, Khater et al. [74] applied the method to fish, meat, and dairy products in Egypt, and de Koster et al. [75] described its broader value in detecting foodborne pathogens and spoilage organisms. MALDI TOF MS has also been adapted for epidemiological analyses. Giraud-Gatineau et al. [76] developed computational pipelines to identify outbreak associated clusters, and Elbehiry et al. [77] validated its use for surveillance of retail meat and poultry in Saudi Arabia. More recently, Feucherolles et al. [18] demonstrated its potential for predicting AMR phenotypes in *Campylobacter* spp., highlighting its emerging role in AMR surveillance.

The strengths of MALDI TOF MS include speed, reproducibility, cost efficiency after initial investment, and suitability for high-throughput analysis [78]. Limitations remain, such as dependence on updated spectral libraries and the inability to directly detect virulence or resistance genes [79,80]. To address these limitations, we integrated real-time PCR with MALDI TOF MS to confirm species reliability and identify genetic resistance determinants. This combined strategy increased confidence in the results and supplied critical AMR information.

AST results showed a high prevalence of MDR, with more than 60% of isolates meeting the MDR definition. *A. baumannii* displayed the highest levels of phenotypic resistance among the species examined; however, resistance to ampicillin and cefotaxime reflects intrinsic resistance and was therefore not considered in the interpretation of acquired multidrug resistance [81]. MDR classification for *A. baumannii* was based solely on antibiotics informative for acquired resistance and should be interpreted with caution because clinically important agents such as carbapenems and ampicillin/sulbactam were not included in the susceptibility testing panel. *S. aureus* isolates demonstrated high resistance to ampicillin (72.1%) and erythromycin (62.8%), with about 35% identified as MRSA, confirming *mecA*-mediated resistance. These findings exceed global pooled estimates [82,83] and are consistent with regional data from Egypt and Iran [84,85]. *E. coli* showed high resistance to ampicillin and trimethoprim sulfamethoxazole, and 43.6% were resistant to cefotaxime, indicating reduced susceptibility to third generation cephalosporins. The detection of *blaTEM*, *blaCTX-M*, and *blaSHV* reflects the presence of ESBL-associated resistance genes; however, phenotypic ESBL confirmation testing was not performed. The detection of *blaTEM*, *blaCTX M*, and *blaSHV* confirmed ESBL presence, matching global observations [86,87]. *Salmonella* spp. exhibited moderate resistance to ampicillin, tetracycline, and streptomycin, in keeping with international reports [88,89,90].

Molecular profiling confirmed phenotypic patterns. In staphylococci, *blaZ* and *mecA* were detected, confirming beta lactamase production and MRSA status [91]. In *A. baumannii*, OXA-type genes including *blaOXA-23*, *blaOXA-24/40*, and *blaOXA-58* were identified; however, their phenotypic impact could not be assessed in the absence of carbapenem susceptibility testing [92,93]. Other determinants such as *tetA*, *tetM*, *aac(6′) Ib*, *aph(3′) IIIa*, *ermB*, and *sul1* confirmed that RTE fast foods in Al Madinah may act as reservoirs of clinically significant resistance genes [94,95].

The detection of high contamination rates and multidrug-resistant phenotypes, together with the presence of clinically relevant resistance genes in *A. baumannii*, has important public health implications, especially in a mass gathering city such as Al Madinah. Antimicrobial resistant foodborne pathogens from local outlets may spread globally through transient populations. Mass gatherings can amplify foodborne events, as shown during the Arba’een pilgrimage [96]. Recent food safety inspections during Hajj 2022 reported variation in compliance, with Al Madinah outlets generally performing better than those in Mecca, especially where staff received training [97]. These findings highlight the need to integrate food safety into One Health frameworks and prioritize hygiene interventions in fast food settings that serve mass gatherings.

## 5. Strengths, Limitations, and Future Directions

This study is supported by several strengths. The integrated design combined culture, MALDI TOF MS, AST, and real-time PCR, allowing both phenotypic and genotypic confirmation of species identity and AMR determinants. This dual approach offered a level of reliability beyond that of single-method workflows. Another strength was the systematic sampling framework, which included international fast food franchises, local restaurants, and informal street vendors across Al-Madinah Al-Munawarah. Because the city serves millions of pilgrims each year, this broad sampling increases the epidemiological relevance of the findings and underscores their importance for local and global public health.

Despite these strengths, several limitations should be noted. First, the reliability of MALDI TOF MS depends on the completeness and updating of spectral libraries, which may lead to misidentification of rare or emerging strains, as described by [37]. Second, while PCR allowed reliable detection of key resistance genes, it was limited to a targeted panel and may have missed other clinically important determinants or mobile genetic elements. Third, the cross-sectional design provided only a single time point, limiting assessment of seasonal or temporal variation. This design also prevented evaluation of changes in contamination levels or AMR profiles over time. This study did not include environmental or handler sampling, such as swabs from equipment, preparation surfaces, or food handlers. This limits interpretation of contamination sources and hygiene compliance. Information on temperature control, storage duration, and post cooking handling was not systematically recorded, preventing correlation of environmental practices with contamination or AMR outcomes.

Fourth, although sampling covered diverse food categories and vendor types, certain subgroups, including fish-based foods and informal street vendors, had smaller sample sizes, limiting the power of subgroup comparisons. Finally, while dendrograms and PCA mapping provided valuable insights into isolate clustering, higher-resolution phylogenetic approaches such as multilocus sequence typing, whole-genome solid nanoparticle-based phylogeny, or cgMLST were not performed. These methods would enable tracking of transmission routes and clonal spread. This study also did not incorporate upstream veterinary or agricultural data, such as livestock sources, meat supply chains, or water quality, which would strengthen One Health interpretation of links between community and clinical AMR reservoirs.

Future work should address these gaps by integrating whole-genome sequencing for detailed characterization of resistance determinants, virulence factors, and clonal lineages. Whole-genome sequencing has been widely applied in One Health AMR surveillance and allows international comparison of isolates and outbreak tracking along the food chain [98,99,100]. Metagenomic sequencing could complement culture-based methods by detecting unculturable or low-abundance pathogens in complex food matrices [69,101].

## 6. Conclusions

This study shows that RTE fast foods in Al-Madinah Al-Munawarah are important vehicles for food-associated bacterial contamination and AMR determinants. *S. aureus* and *E. coli* predominated, and *A. baumannii* was detected in this setting. The high proportion of MRSA and the detection of ESBL-associated resistance genes in *E. coli* highlight potential public health concerns associated with these foods, particularly in a global mass gathering city. The detection of *A. baumannii* and associated resistance genes is reported descriptively and should be interpreted cautiously, as this organism is not a classical foodborne pathogen and phenotypic carbapenem susceptibility testing was not performed. By integrating MALDI TOF MS with real-time PCR, we established a rapid, reliable, and scalable diagnostic workflow that confirmed species identity and revealed critical resistance determinants. This approach reduced diagnostic turnaround to less than 24 h compared with 48 to 72 h for conventional methods. When applied within its methodological limits, embedding such workflows into One Health surveillance and food safety monitoring may support timely risk assessment, strengthen outbreak response, and enhance preparedness against AMR dissemination during mass gatherings.

## Figures and Tables

**Table 1:** Real-time PCR targets, primer sequences, amplicon sizes, and references used for species confirmation and AMR gene detection.

Bacterial Species	Target Gene	Primer Sequence (5′ → 3′)	Amplicon Size (bp)	Function	Ref.
*E. coli*	*uidA*	F: TGGTAATTACCGACGAAAACGGC R: ACGCGTGGTTACAGTCTTGCG	147	Species confirmation	[41]
*S. aureus*	*nuc*	F: GCGATTGATGGTGATACGGTT R: AGCCAAGCCTTGACGAACTAAAGC	279	Species confirmation	[42]
*Salmonella* spp.	*invA*	F: GTGAAATTATCGCCACGTTCGGGCAA R: TCATCGCACCGTCAAAGGAACC	284	Species confirmation	[43]
*A. baumannii*	*blaOXA-51*-like	F: TAATGCTTTGATCGGCCTTG R: TGGATTGCACTTCATCTTGG	353	Species confirmation	[44]
*E. coli, Salmonella*	*blaTEM*	F: ATGAGTATTCAACATTTCCGTG R: TTACCAATGCTTAATCAGTGAG	858	β-lactam resistance	[45]
	*blaCTX-M*	F: ATGTGCAGYACCAGTAARGTKATGGC R: TGGGTRAARTARGTSACCAGAAYCAGCGG	593	β-lactam resistance	[46]
	*blaSHV*	F: ATGCGTTATATTCGCCTGTG R: TGCTTTGTTATTCGGGCCAA	747	β-lactam resistance	[45]
	*tetA*	F: GGTTCACTCGAACGACGTCA R: CTGTCCGACAAGTTGCATGA	577	Tetracycline resistance	[47]
	*tetM*	F: GTGGACAAAGGTACAACGAG R: CGGTAAAGTTCGTCACACAC	406	Tetracycline resistance	[47]
	*aac(6′)-Ib*	F: TTGCGATGCTCTATGAGTGGCTA R: CTCGAATGCCTGGCGTGTTT	482	Aminoglycoside resistance	[48]
	*aph(3′)-IIIa*	F: GGCTAAAATGAGAATATCACCGG R: CTTGTCGTGATAAGCCAGTCC	523	Aminoglycoside resistance	[49]
	*sul1*	F: CGCACCGGAAACATCGCTGCAC R: TGAAGTTCCGCCGCAAGGCTCG	432	Sulfonamide resistance	[50]
*S. aureus*	*mecA*	F: AAAATCGATGGTAAAGGTTGGC R: AGTTCTGCAGTACCGGATTTGC	533	Methicillin resistance	[51]
	*blaZ*	F: ACTTCAACACCTGCTGCTTTC R: TGACCACTTTTATCAGCAACC	173	β-lactam resistance	[52]
	*ermB*	F: GAAAAGGTACTCAACCAAATA R: AGTAACGGTACTTAAATTGTTTAC	639	Macrolide resistance	[53]
*A. baumannii*	*blaOXA-23*-like	F: GATCGGATTGGAGAACCAGA R: ATTTCTGACCGCATTTCCAT	501	Carbapenem resistance	[54]
	*blaOXA-24/40*-like	F: GGTTAGTTGGCCCCCTTAAA R: AGTTGAGCGAAAAGGGGATT	246	Carbapenem resistance	[55]
	*blaOXA-58*-like	F: AAGTATTGGGGCTTGTGCTG R: CCCCTCTGCGCTCTACATAC	599	Carbapenem resistance	[46]

**Table 2:** Distribution of RTE fast food samples by outlet type and food category in Al-Madinah Al-Munawarah (n = 300).

Variable	Category	n	%
Outlet type	International franchises	75	25.0
	Local restaurants	75	25.0
	Street vendors (informal/semi-formal)	150	50.0
Food category	Chicken shawarma	40	13.3
	Beef shawarma	40	13.3
	Burgers (chicken and beef)	50	16.7
	Fried chicken	50	16.7
	Sandwiches (other than shawarma/burgers)	60	20.0
	Fresh salads	60	20.0

**Table 3:** Estimated prevalence of bacterial isolates recovered from RTE fast food samples in Al-Madinah Al-Munawarah (n = 300).

Food Category	*E. coli*		*S. aureus*		*Salmonella* spp.		*A. baumannii*		Total Positive Samples	
	No.	%	No.	%	No.	%	No.	%	No.	%
Chicken shawarma	9	22.5	10	25.0	3	7.5	2	5.0	24	60.0
Beef shawarma	8	20.0	9	22.5	3	7.5	2	5.0	22	55.0
Burgers	10	20.0	11	22.0	4	8.0	3	6.0	28	56.0
Fried chicken	5	10.0	6	12.0	9	18.0	7	14.0	27	54.0
Sandwiches	4	6.7	5	8.3	5	8.3	3	5.0	17	28.3
Fresh salads	3	5.0	2	3.3	3	5.0	3	5.0	11	18.3
Total (n = 300)	39	13.0	43	14.3	27	9.0	20	6.7	129	43.0

**Table 4:** MALDI-TOF MS identification of bacterial isolates recovered from RTE fast foods in Al-Madinah Al-Munawarah (n = 129).

Bacterial Species	No. of Isolates	Secure Species-Level ID (≥2.0)	Genus-Level ID (1.7–1.99)	Species-Level Identification Rate (%)
*S. aureus*	43	42	1	97.7
*E. coli*	39	38	1	97.4
*Salmonella* spp.	27	25	2	92.6
*A. baumannii*	20	19	1	95.0
Total	129	124	5	96.1

**Table 5:** AMR profiles of bacterial isolates recovered from RTE fast foods in Al-Madinah Al-Munawarah (n = 129).

Bacterial Species	AMP (%)	FOX (%)	CTX (%)	GEN (%)	STR (%)	TET (%)	DOX (%)	CIP (%)	LEV (%)	CHL (%)	ERY	SXT (%)	MDR Prevalence	Statistical Significance (*p*-Value)
*S. aureus* (n = 43)	72.1	34.9	NA	NA	NA	44.2	NA	39.5	NA	0.0	62.8	NA	65.1%	Erythromycin higher vs. others (*p* < 0.05)
*E. coli* (n = 39)	69.2	NA	43.6	NA	NA	NA	NA	33.3	30.8	NA	NA	61.5	61.5%	Ampicillin resistance higher vs. *Salmonella* (*p* < 0.01)
*Salmonella* spp. (n = 27)	40.7	NA	NA	NA	25.9	29.6	NA	NA	0.0	0.0	NA	NA	37.0%	Lowest MDR compared to others (NS)
*A. baumannii* (n = 20)	NA	NA	NA	55.0	NA	>70.0	45.0	>70.0	NA	NA	NA	NA	85.0%	MDR highest vs. all species (*p* < 0.001)

Abbreviations: AMP, ampicillin; FOX, cefoxitin; CTX, cefotaxime; GEN, gentamicin; STR, streptomycin; TET, tetracycline; DOX, doxycycline; CIP, ciprofloxacin; LEV, levofloxacin; CHL, chloramphenicol; ERY, erythromycin; SXT, trimethoprim–sulfamethoxazole; MDR, multidrug resistance; NA, not applicable.

**Table 6:** Frequency of AMR genes detected among bacterial isolates from RTE fast foods in Al-Madinah Al-Munawarah, with statistical comparisons across bacterial species.

Bacterial Species	Resistance Gene	*n/N*	Frequency (%)	Associated Resistance	*p*-Value
*S. aureus* (n = 43)	*mecA*	14/43	32.6	Methicillin resistance	<0.05
	*blaZ*	30/43	69.8	Penicillin resistance	<0.01
*E. coli* (n = 39)	*blaTEM*	22/39	56.4	β-lactams	<0.01
	*blaCTX-M*	16/39	41.0	Extended-spectrum cephalosporins	<0.05
	*blaSHV*	12/39	30.8	β-lactams	NS
*Salmonella* spp. (n = 27)	*tetA*	9/27	33.3	Tetracyclines	NS
	*sul1*	7/27	25.9	Sulfonamides	NS
*A. baumannii* (n = 20)	*blaOXA-51*-like	20/20	100.0	Intrinsic carbapenem resistance	<0.001
	*blaOXA-23*	14/20	70.0	Carbapenem resistance	<0.01
	*blaOXA-24/40*	9/20	45.0		<0.05
	*blaOXA-58*	5/20	25.0		NS

Abbreviations: *n/N*, number of positive isolates (*n*) out of the total number tested (*N*); NS, not significant; *bla*CTX-M, extended-spectrum β-lactamase gene; *bla*SHV, extended-spectrum β-lactamase gene; *bla*Z, penicillinase gene; *mecA*, methicillin resistance gene; *tetA*, tetracycline resistance gene; *sul1*, sulfonamide resistance gene; *bla*OXA-51-like, intrinsic OXA carbapenemase gene (*Acinetobacter baumannii*); *bla*OXA-23, *bla*OXA-24/40, and *bla*OXA-58, acquired carbapenemase genes in *A. baumannii*.

**Table 7:** Concordance between phenotypic resistance (AST) and resistance gene carriage detected by real-time PCR among bacterial isolates.

Bacterial Species	Antibiotic Class	Phenotypic Resistance (%)	Resistance Gene(s)	Gene Prevalence (%)	Concordance (%)	Statistical Significance (*p*-Value)
*E. coli* (n = 39)	β-lactams (ampicillin, cefotaxime)	69.2 (AMP), 43.6 (CTX)	*blaTEM*, *blaCTX-M*, *blaSHV*	56.4, 41.0, 30.8	82.0	*p* < 0.01
*S. aureus* (n = 43)	β-lactams (penicillin, methicillin)	72.1 (AMP), 34.9 (FOX)	*blaZ*, *mecA*	69.8, 32.6	88.0	*p* < 0.001
*Salmonella* spp. (n = 27)	Tetracyclines, sulfonamides	29.6 (TET), 25.9 (SUL)	*tetA*, *sul1*	33.3, 25.9	84.0	*p* < 0.05
*A. baumannii* (n = 20)	Carbapenems, cephalosporins	>70.0 (AMP, CTX), >70.0 (CIP)	*blaOXA*-23, *blaOXA*-24/40, *blaOXA*-58	70.0, 45.0, 25.0	85.0	*p* < 0.001

## Data Availability

The raw data supporting the conclusions of this article will be made available by the authors on request.
